# Progress on the Effects of Permissive Hypercapnia on the CNS During the Intraoperative Period: A Narrative Review

**DOI:** 10.7759/cureus.68087

**Published:** 2024-08-29

**Authors:** Ya-zhi Xi, Xiao-yu Jia, Xue-lian Wei, Qing-he Zhou

**Affiliations:** 1 Anesthesiology, Zhejiang Chinese Medical University, Hangzhou, CHN; 2 Anesthesiology and Pain Medicine, The Affiliated Hospital of Jiaxing University, Jiaxing, CHN

**Keywords:** general anesthesia, surgery, intraoperative period, central nervous system, permissive hypercapnia

## Abstract

Previous experimental findings and clinical evidence have shown the important role of carbon dioxide (CO_2_) in regulating cerebral vascular tension. CO_2_ can affect the CNS through various mechanisms. With factors such as patient physiology or surgical interventions potentially causing increased arterial partial pressure of carbon dioxide (PaCO_2_) levels during mechanical ventilation in general anesthesia, it is important to explore the potential risks or benefits of intraoperative permissive hypercapnia on brain function. In November 2023, we conducted a thorough review of PubMed to establish the article outline. Articles that were non-English or repetitive were eliminated. We collected information on the year, topic, key findings, and opinions of each article. This review not only comprehensively summarizes the factors that contribute to the elevation of intraoperative PaCO_2_, but also explores the impact of fluctuations in PaCO_2_ levels on the CNS and the underlying mechanisms involved. At the same time, this article provides our understanding of the potential clinical significance of actively regulating PaCO_2_ levels. In addition, we propose that the aspects of permissive hypercapnia can be further studied to provide a reliable basis for clinical decision-making. The effects of permissive hypercapnia on the CNS remain a topic of debate. Further prospective randomized controlled studies are needed to determine if permissive hypercapnia can be safely promoted during mechanical ventilation in general anesthesia.

## Introduction and background

Carbon dioxide (CO_2_) is a byproduct of cellular metabolism and plays a crucial role in various physiological processes, including the transportation of oxygen, maintenance of acid-base balance, and regulation of blood perfusion in the brain and other organs. The arterial partial pressure of carbon dioxide (PaCO_2_) reflects the concentration of CO_2 _dissolved in the plasma and is primarily influenced by the combination of inhaled CO_2_ concentration, alveolar ventilation, and cellular CO_2_ production [1]. The regulation of PaCO_2_ is governed by negative feedback mechanisms involving both central and peripheral chemoreceptors. It is widely recognized that CO_2_ plays a significant role in regulating cerebral vascular tension. The normal range of PaCO_2_ (at sea level and 37 °C) falls between 35 and 45 mmHg (1 mmHg = 0.133 kPa). However, when PaCO_2_ exceeds 45 mmHg, a condition known as hypercapnia, it can potentially impact the CNS through cerebral microcirculation and metabolism.

Permissive hypercapnia was initially proposed and implemented in the management of acute lung injury and diseases associated with acute respiratory distress syndrome [2]. It means the implementation of a low tidal volume ventilation approach that permits a modest elevation in the level of PaCO_2_, concurrently enabling a certain level of acidosis. This approach effectively mitigates the risk of ventilator-associated lung injury resulting from excessive lung expansion caused by high tidal volume and elevated airway pressure [3-5].

Given sufficient evidence of permissive hypercapnia in reducing lung complications, many studies in recent years have attempted to explore other benefits of permissive hypercapnia, such as reducing inflammatory reactions and providing multiple organ protection [6-8]. However, in long-term follow-up of coma patients who underwent resuscitation after cardiac arrest outside the hospital, it was not found that mild hypercapnia improved the prognosis of the nervous system compared to normal blood CO_2_ levels [9]. As the brain serves as the center of life, intraoperative cerebral protection is particularly important. Therefore, it is of great significance to study the potential risks or benefits of permissive hypercapnia on brain function under the implementation of lung protective ventilation strategies during operation. End-expiratory carbon dioxide pressure (PetCO_2_) has a certain correlation with PaCO_2_ [10]. During mechanical ventilation under general anesthesia, the target level of PetCO_2_ is achieved by adjusting the tidal volume and respiratory rate, making permissive hypercapnia easy to implement.

Most of the previous reviews on permissive hypercapnia and its effects on the brain only focus on the cerebrovascular, which is not comprehensive enough. Based on some clinical studies published in recent years and related research results from our team, this narrative review aims to investigate the effects of permissive hypercapnia on the CNS, which can provide a reference for clinicians to find the best perioperative management scheme.

## Review

Factors that cause hypercapnia during the intraoperative period

The fluctuation of PaCO_2_ is affected by different types of surgery. For example, laparoscopic surgery commonly employs CO_2_ for the purpose of establishing pneumoperitoneum, and the CO_2_ pressure gradient of the abdominal cavity-blood intervals and the high solubility of CO_2_ in the blood cause CO_2_ to quickly absorb through the peritoneum and cause hypercapnia [11]. At the same time, clinical observations have found that in some surgeries, such as kidney, prostate, and inguinal hernia procedures, which require creating artificial gaps or pelvic lymphadenectomy with large areas of loose tissue, the peritoneum has a higher CO_2_ absorption capacity, resulting in a more noticeable increase in PaCO_2_ levels. In addition, thoracic surgery is prone to causing ventilation/blood flow ratio (V/Q) dysfunction during one-lung ventilation, which affects the exchange between alveolar and arterial blood, resulting in CO_2_ retention [3].

It is generally believed that surgical operational errors may cause complications such as subcutaneous emphysema, pneumothorax, or air embolism. Additionally, respiratory depression can occur during intravenous anesthesia without endotracheal intubation. High metabolism resulting from surgical stress, along with high-risk individuals - such as those who are obese or critically ill - can also contribute to elevated PaCO_2_ levels.

Effect of permissive hypercapnia on the CNS

**Figure 1:**
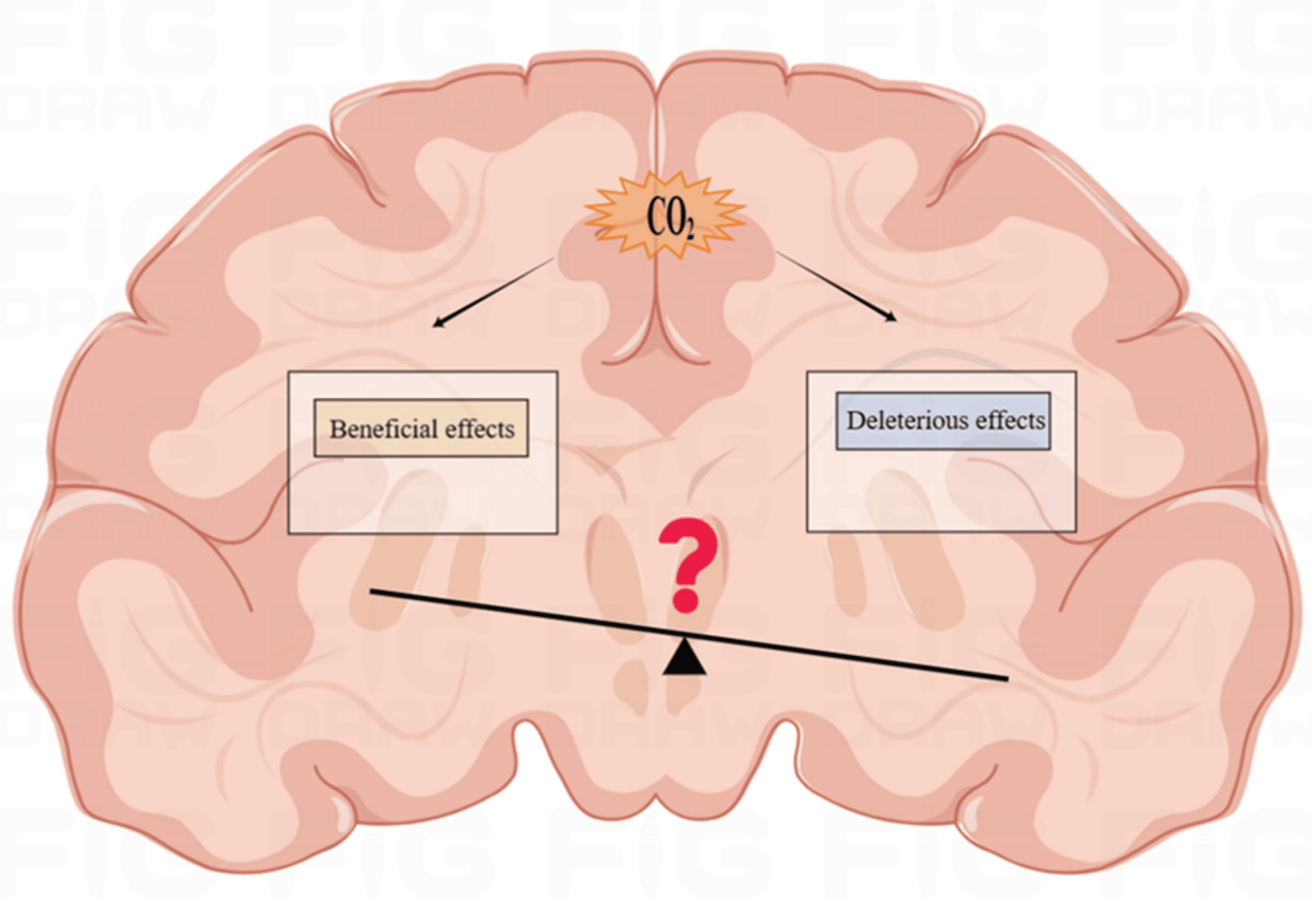
Uncertain effects of CO₂ on the CNS: potential beneficial or deleterious effects Original illustration by Ya-zhi Xi

The impact of permissive hypercapnia on the CNS is currently uncertain (Figure 1).

Some studies suggest that hypercapnia has a neuroprotective effect and can reduce the occurrence of cognitive impairment (Table 1) [12-19].

**Table 1:** Summary of benefits of permissive hypercapnia for CNS BBB: blood-brain barrier; CBF: cerebral blood flow; NSE: neuron-specific enolase; PD: Parkinson’s disease; TH: therapeutic hypercapnia; THMH: targeted therapeutic mild hypercapnia; TMH: targeted mild hypercapnia

Study	Year	Model	Effects on CNS	CO_2_
Yang et al. [12]	2019	Rat	TH ameliorated BBB damage and improved the neurologic outcome in a rat model of lateral fluid percussion injury.	80-100 mmHg
Vannucci et al. [13]	1997	Rat	During hypoxia-ischemia in the immature rat, CBF is better preserved during normocapnia and hypercapnia; the greater oxygen delivery promotes cerebral glucose utilization and oxidative metabolism for optimal maintenance of tissue high-energy phosphate reserves. Inhibition of glutamate secretion into the synaptic cleft and its attenuation of N-methyl-D-aspartate receptor activation would further protect the hypercapnic animal from hypoxic-ischemic brain damage.	55 mmHg
Wong et al. [15]	2020	Human	TMH during major surgery was associated with a stable increase in rSO_2_ from the baseline. Furthermore, postoperative delirium was lower in the TMN group.	45-55 mmHg
Tregub et al. [16]	2023	Rat	The exposure models where permissive hypercapnia and normobaric hypoxia were combined also had the most pronounced inhibitory effects on apoptotic signaling pathways, resulting in the maximum neuroprotective effect.	50 mmHg
Eastwood et al. [17]	2016	Human	THMH was feasible, appeared safe, and attenuated the release of NSE compared with targeted normocapnia.	50-55 mmHg
Tao et al. [18]	2013	Rat	TH preserves brain tissue and promotes functional neurological recovery through antiapoptotic mechanisms.	80-100 mmHg
Nadeev et al. [19]	2022	Mouse	High CO_2_ inhalation presumably initiates mitophagy via transient brain acidification and can treat PD-like symptoms in a rodent rotenone model of PD.	Three times a week, animals were placed in a closed glass cylinder, filled with 20% CO_2_ atmosphere for two minutes.

However, other studies have found that the presence of hypercapnia is related to the occurrence and development of cognitive impairment (Table 2) [20-26].

**Table 2:** Summary of harms of permissive hypercapnia for CNS BBB: blood-brain barrier; pH: potential of hydrogen; VLBW: very low birth weight

Study	Year	Model	Effects on CNS	CO_2_
Ding et al. [20]	2020	Rat	Hypercapnia-induced IL-1β overproduction in the hypoxemic blood may decrease tight junctional protein expression in cerebrovascular endothelial cells via the IL-1R1/p-IRAK-1 pathway, further disrupting the BBB integrity, and eventually resulting in increased BBB permeability.	Exposed to 5% CO_2_
Kaiser et al. [21]	2005	Human	The autoregulatory slope increases with increasing PaCO_2_, suggesting that autoregulation in ventilated VLBW infants during the first week of life may be associated with increased vulnerability to brain injury.	PCO_2_ >45 mmHg
Ding et al. [22]	2018	Rat	Hypercapnia-induced IL-1β overproduction via activating the NLRP3 inflammasome by hypoxia-activated microglia may augment neuroinflammation, increase neuronal cell death, and contribute to the pathogenesis of cognitive impairments.	Exposed to CO_2_ concentrations of 5% of the gas mixture to maintain pH at 7.20-7.25
Tiruvoipati et al. [23]	2018	Human	Hypercapnic acidosis was associated with an increased risk of hospital mortality in patients with cerebral injury. Hypercapnia, when compensated to a normal pH during the first 24 hours of intensive care unit admission, may not be harmful in mechanically ventilated patients with cerebral injuries.	PCO_2_ >45 mmHg
Ding et al. [25]	2020	Rat	Hypercapnia promotes microglial pyroptosis via inhibiting mitophagy in hypoxemic adult rats.	Exposed to 5% CO_2_ to maintain the pH of arterial blood at 7.20-7.25
Wang et al. [26]	2015	Human	The presence of intraoperative hypercapnia appears to be associated with the development of postoperative delirium in geriatric patients after orthopedic surgery.	Not mentioned

The following will elaborate on the impact of hypercapnia on the CNS and its clinical application through monitoring and evaluation of indicators such as CNS biomarkers, EEG, and regional cerebral oxygen saturation (rSO_2_).

CNS Biomarkers

Neuromarkers related to brain injury, which are specifically expressed in specific nerve cells, can be used to confirm the localization of target cells. Neuron-specific enolase (NSE) is a protein found in neurons and neuroendocrine cells. NSE is released from the cytoplasm and enters the cerebrospinal fluid and blood when neurons are damaged or necrotic [27,28]. A randomized controlled study showed that mild hypercapnia induced after out-of-hospital cardiac arrest (OHCA) can reduce serum NSE levels, and after six months, the hypercapnia group showed better functional recovery compared to the control group [17]. Brain-derived neurotrophic factor (BDNF), a protein with neurotrophic effects, is related to the protection and repair of the nervous system [29]. Experiments on animals have revealed that intermittent hypercapnia combined with hypoxia can enhance the production of BDNF protein [30]. Neurofilament light chain (NFL) is only found in the axons and fibers of neurons and is usually increased in cases of brain damage or neurological diseases. It is highly specific and can be used to indicate neurological damage [31]. Despite the high accuracy of the NFL in predicting the prognosis of OHCA, there is no evidence to suggest a correlation between different levels of arterial blood CO_2_ concentration and NFL levels [32]. However, our team revealed that the NFL concentration demonstrated a declining trend in the hypercapnia group one day postoperatively, but there was no statistical difference between the two groups in a recent prospective randomized controlled study.

EEG

EEG captures, amplifies, and records the spontaneous bioelectric signals generated in the brain by using electrodes. EEG can predict the impact of hypercapnia on the CNS. Studies have shown that hypercapnia can lead to an increase in slow wave frequency (δ wave) and a decrease in fast wave frequency bands (α wave, β wave, and γ wave) [33,34]. This shift in the EEG spectrum indicates a general slowing down of the electrical activity of the brain’s neurons, reflecting an inhibitory state of neurons. Additionally, the brain’s metabolic rate decreases, the demand for oxygen and nutrients by neurons is reduced, and the brain’s sensitivity to hypoxia is reduced, providing a protective effect on brain function.

rSO_2_

Noninvasive near-infrared spectroscopy measurement is used to assess the oxygen transport and consumption in the frontal brain region through the rSO_2_, which is the mixed blood oxygen saturation of the cerebral microcirculation. It was observed by Park et al. [14] that keeping mild hypercapnia during laparoscopic examination could result in higher rSO_2_ levels for patients. Similarly, a randomized controlled study showed that rSO_2_ levels rose significantly from the beginning to the end of the operation in those with mild hypercapnia, which was associated with a decrease in the rate of postoperative cognitive impairment [15]. The impact of hypercapnia on cerebral oxygen saturation is complex and may be influenced by various factors, such as cardiac output, hemoglobin affinity for oxygen, cerebral autoregulation, and the ratio of cerebral arterial to venous blood volume [35,36].

The related mechanism of hypercapnia affecting the CNS

Cerebral Microcirculation

As a highly oxygen-consuming organ, the brain only accounts for 2% of the body’s weight. However, under normal circumstances, the blood flow of the entire brain accounts for about 15% of the total cardiac output. Cerebral blood flow (CBF) depends on the ratio of cerebral perfusion pressure (CPP) to cerebral vascular resistance (CVR) (CBF = CPP/CVR). CPP is the difference between the mean arterial blood pressure (MAP) and the intracranial pressure (ICP) (CPP = MAP-ICP). PaCO_2_ is the most effective regulatory factor for CBF, and even small fluctuations can lead to significant changes in CBF. Hypercapnia can affect cerebral microcirculation in the following aspects:

Firstly, the automatic regulation function of CBF affects cerebral microcirculation. The automatic regulation function of CBF was first proposed by Lassen [37], which is the mechanism of keeping CBF steady when perfusion pressure fluctuates, showing the correlation between CBF and CPP. At present, it is believed that the automatic regulation curve of CBF is composed of three parts: the lower limit, the platform, and the upper limit, and is regulated by multiple non-perfusion pressure-related mechanisms, with CO_2_ playing a significant role. Hypercapnia can lead to the following changes in the CBF autoregulation curve: the lower limit of the curve shifts to the right, the upper limit shifts to the left, and the autoregulation plateau becomes narrower [38]. The extent of the change is dependent on the level of arterial CO_2_ [39,40]. Kaiser et al. [21] found that in premature babies with low birth weight, the capacity for self-regulation of CBF gradually impairs as the level of hypercapnia increases. At the same time, there are differences in the threshold of automatic regulation of CBF under different anesthetic drugs under hypercapnic conditions. The results of McCulloch et al. [41] indicate that when under hypercapnia, the average threshold for impaired automatic regulation of CBF is (56 ± 4) mmHg with sevoflurane anesthesia and (61 ± 4) mmHg with propofol anesthesia. Therefore, the dynamic changes in CBF autoregulation function should be comprehensively considered during surgery based on the patient’s levels of hypercapnia and the type of anesthetic medication used.

Secondly, the reactivity of cerebral blood vessels affects cerebral microcirculation. The reactivity of cerebral blood vessels refers to the ability of resistance vessels to vasodilate or contract in response to changes in the arterial PaCO_2_. The mechanism may be that CO_2_ promotes the release of potassium, calcium, nitric oxide, cyclic nucleotides, prostaglandins, and other mediators from cerebral microvascular endothelial cells, which regulate vascular smooth muscle tension [42,43]. Through intracellular second messengers like cGMP/cAMP, mediators activate K-ATP and K-Ca channels, which in turn lower intracellular Ca2þ and cause vasodilation [44]. Recent studies using blood oxygen level-dependent brain functional imaging signals suggest that there is a curvilinear relationship between CBF and PaCO_2_. When PaCO_2 _levels increase within the range of vasodilation reserve, it leads to an increase in CBF. However, when PaCO_2_ continues to rise, the local vasodilation of the brain reaches its limit, and the sustained vasodilation response in other regions will trigger “blood theft,” causing a redistribution of CBF, which is mainly concentrated in the brainstem, ventricle, and brain surface [45,46].

In addition, ICP has a significant impact on cerebral microcirculation. ICP refers to the pressure inside the skull, determined by the volume of the cranial contents (blood, brain, and cerebrospinal fluid). CO_2_ is a key factor affecting ICP. Research has shown that the diameter of the optic nerve sheath can reflect changes in ICP, which is positively correlated with PaCO_2_ [47]. Hypercapnia may mainly increase intracranial blood volume by enlarging blood vessels and increasing CBF, thereby increasing ICP. A retrospective study has found that hypercapnia can raise ICP in critically ill patients within 24 hours of admission, even with complete brain autoregulation, resulting in secondary brain injury and a worse clinical prognosis [23].

Interestingly, there is controversy over whether CO_2_ has a direct effect on cerebral blood vessels. Animal studies have demonstrated that changes in CO_2_ and the potential of acidity (pH) can directly affect the conductivity of potassium ions, thereby changing the tension of cerebral arteries [48]. On the other hand, research shows that hydrogen ions serve as both internal and external messengers, and PaCO_2_ adjusts cerebral vessels by changing pH [49,50]. Furthermore, a study has also found that there is a complex relationship between CBF and PCO_2_ HCO^3-^ and pH; any attempt to modify the acid-base balance around cerebral blood vessels can have an impact on CBF [51].

Brain Metabolism

The oxygen consumption of adult brain tissue is 20% of that of the whole body, so meeting the demands of normal brain metabolism is crucial for maintaining brain function. The following will elaborate on the impact of hypercapnia on brain metabolism from the perspectives of the cerebral metabolic rate of oxygen (CMRO_2_), excitatory amino acid neurotransmitters, and mitochondrial metabolic mechanisms.

Firstly, hypercapnia can affect the cerebral metabolic rate of oxygen. CMRO_2_ refers to the rate at which the brain consumes oxygen at a specific time, which is used to measure brain activity and metabolic status. As an indicator of brain energy homeostasis, the brain oxygen metabolism rate has been studied multiple times in the field of hypercapnia, but the results of these studies are not consistent. Abou-Arab et al. [52] found that intraoperative hypercapnia in patients undergoing cardiac surgery did not affect CMRO_2_. Zhou et al. [53] observed that hypercapnia, caused by excessive ventilation in intensive care patients, increased CMRO_2_. However, Deckers et al. [54] found that CMRO_2_ decreased under permissive hypercapnia. At the same time, studies have also shown that hypercapnia can inhibit glycolysis and cell metabolism by causing pH-mediated hypercapnic acidosis, thereby reducing brain oxygen metabolism [55,56]. This suggests that the application of permissive hypercapnia may have a certain neuroprotective effect under hypoxic conditions.

Secondly, hypercapnia can also affect the release of excitatory amino acid neurotransmitters. Glutamate, the most abundant neurotransmitter in the CNS, is found in most neurons and synapses. Its increase can quickly trigger the production of c-Fos protein, leading to excitatory toxicity of neurons and ultimately neuronal death. According to Vannucci et al. [13], the concentration of glutamate in the cerebrospinal fluid of young rats exposed to hypercapnia conditions was lower than that of rats in hypocapnia and normocapnia conditions. This suggests that hypercapnia may protect the CNS by inhibiting the secretion of glutamate into synaptic gaps, reducing the activation of N-methyl-D-aspartate receptors, and alleviating damage caused by free radicals.

In addition, hypercapnia can affect mitochondrial metabolism. Mitochondria are important oxidative metabolic centers within cells and have attracted much attention in diseases related to energy metabolism. On the one hand, research conducted on rats revealed that hypercapnia can activate mitochondrial autophagy through a rapid process of brain acidification, which could be beneficial for treating Parkinson’s-like symptoms [19]. At the same time, hypercapnia can reduce the levels of proteins such as caspase-3, apoptosis-inducing factor, and Bax through anti-apoptotic mechanisms and increase the concentration of Bcl-2, thereby protecting brain tissue and promoting the recovery of neural function [16,18]. However, on the other hand, studies have shown that the inhibition of mitochondrial function caused by hypercapnia can promote microglial cell apoptosis under hypoxic conditions and enhance the release of inflammatory factors such as IL-1β and IL-18, leading to the exacerbation of neuroinflammation and promoting neuronal cell death, thus participating in the occurrence of cognitive dysfunction [22,25].

Moreover, accumulating data show that the permeability of the blood-brain barrier (BBB) serves as a key factor in mediating secondary brain damage; thus, it is critical to investigate modifications in the BBB to gain insight into the effects of hypercapnia on the CNS [57-59]. There is debate surrounding the influence of hypercapnia on the BBB. On the one hand, therapeutic hypercapnia ameliorated BBB damage and improved the neurologic outcome in a rat model of lateral fluid percussion injury [12]. On the other hand, hypercapnia-induced IL 1β overproduction in the hypoxemic blood may decrease tight junctional protein expression in cerebrovascular endothelial cells via the IL 1R1/p IRAK 1 pathway, further disrupting the BBB integrity, eventually resulting in increased BBB permeability [20]. Other mechanisms, such as free radical-induced damage [60,61] and the inflammatory response [62,63], also play a role in the effect on brain function.

Other potential applications of permissive hypercapnia

With pulmonary complications increasingly valued, more and more studies advocate the implementation of lung protective ventilation strategies during surgery [64,65]. The accepted approach is to use low tidal volume ventilation with appropriate positive end-expiratory pressure, which can result in an increase in PaCO_2_. Therefore, the application of permissive hypercapnia is receiving more attention from anesthesiologists.

Interestingly, recent studies have revealed that hypercapnia, when combined with excessive ventilation, can help patients recover faster from less soluble anesthetics such as sevoflurane and desflurane during the recovery period. The mechanism may be an increase in cardiac output, relaxation of cerebral arterial smooth muscle, and an increase in CBF caused by hypercapnia, which accelerates the clearance of volatile anesthetics from brain tissue [66-68]. A subsequent study, however, has proposed a different viewpoint, stating that in the case of hypercapnia in anesthetized patients during awakening, there is no statistically significant difference in awakening time compared to the control group [69], but the specific mechanism remains unclear.

In summary, the effect of intraoperative permissive hypercapnia remains unclear in perioperative management. However, it is not recommended for those with impaired intracranial compliance or risk of intracranial hypertension due to the increased ICP it causes. In addition, hypercapnia can increase sympathetic nervous tension and affect the left ventricular function. Therefore, continuous monitoring of end-expiratory CO_2_ levels is essential for the treatment and care of high-risk surgical patients. Whether permissive hypercapnia should be considered an acceptable effect on the body still needs further validation in the human body to ensure its safety and efficacy.

The duration and target levels of hypercapnia are critical factors for clinicians to consider. Short-term or mild hypercapnia, which increases CBF, generally has a compensatory effect that helps maintain oxygen supply to brain tissues. However, prolonged or severe hypercapnia can increase ICP and even alter pH levels within brain cells, affecting neuronal metabolic activity and function. Therefore, further clinical trials are needed to validate the impact of the duration and levels of hypercapnia on brain function.

## Conclusions

Based on our knowledge of this field and our clinical findings, we regard the topic of “the effect of intraoperative hypercapnia on the CNS” as interesting, reasonable, and clinically important. Although permissive hypercapnia was proposed a century ago, current research mainly focuses on the protection of the lungs. However, it is important to note that hypercapnia can also have an impact on cerebral microcirculation and brain metabolism. The effects of intraoperative permissive hypercapnia on the CNS remain unclear. Future research should use various monitoring techniques, such as CNS biomarkers, EEG, and cerebral oxygen saturation, to analyze the changes in neural function caused by permissive hypercapnia from multiple dimensions. At the same time, we also need to consider the long-term impacts of permissive hypercapnia on patients, including cognitive function and quality of life, to get an accurate assessment of its effect on the CNS. Moreover, additional studies must be conducted to explore the potential mechanisms of permissive hypercapnia on brain function in order to gain a deeper insight into related molecular mechanisms, signaling pathways, and regulatory factors. This will provide a more reliable basis for clinical decision-making.
